# P65 Implementation of antimicrobial stewardship in Bangladesh healthcare settings: a systematic review of challenges and opportunities

**DOI:** 10.1093/jacamr/dlaf230.072

**Published:** 2025-12-04

**Authors:** Shumonto Mowla Chowdhury, Hyacinth Ukuhor, Rasha Abdelsalam Elshenawy

**Affiliations:** Department of Medicine, School of Health, Medicine and Life Sciences, University of Hertfordshire, Hatfield AL10 9AB, UK; Department of Medicine, School of Health, Medicine and Life Sciences, University of Hertfordshire, Hatfield AL10 9AB, UK; Department of Medicine, School of Health, Medicine and Life Sciences, University of Hertfordshire, Hatfield AL10 9AB, UK

## Abstract

**Background:**

Antimicrobial resistance (AMR) is a critical global health threat, responsible for 1.27 million deaths in 2019, with projections rising to 1.91 million annually by 2050.^1^ Antimicrobial stewardship (AMS) is vital for optimizing antibiotic use and limiting resistance.^2^ Low- and middle-income countries carry a disproportionate burden, with Bangladesh reporting 26 200 AMR-attributable deaths in 2019.^3^ Despite the launch of a National Action Plan (2023–2028), substantial hospital-level challenges remain, including limited stewardship implementation, inconsistent prescribing and underdeveloped evidence-based strategies.^4^

**Objectives:**

This study aims to investigate the opportunities and challenges associated with AMS implementation in Bangladeshi health care settings across different levels and provide evidence-based recommendations.

**Methods:**

This systematic review adhered to PRISMA 2020 guidelines. Searches of MEDLINE, CINAHL, SCOPUS, Cochrane Library and Google Scholar were conducted in August 2024 without date restrictions, guided by the Population, Exposure and Outcomes (PEO) framework. Eligible studies addressed antimicrobial stewardship (AMS) implementation challenges, prescribing attitudes, or AMS interventions in Bangladesh. Quality appraisal used Critical Appraisal Skills Programme (CASP) checklists. Data extraction employed standardized forms, with mixed-methods synthesis combining quantitative and qualitative thematic analysis. Zotero, Mendeley and Microsoft Excel supported reference management and analysis. Ethical approval was not required.

**Results:**

Fourteen studies encompassing 34 097 participants were analysed. Principal implementation barriers included absent institutional AMS policies (*n*=6), entrenched empirical prescribing practices (*n*=5), inadequate diagnostic infrastructure (*n*=4) and lack of dedicated AMS teams (*n*=3). Key facilitators comprised surveillance systems (*n*=6), structured training programmes (*n*=5) and leadership commitment (*n*=3). Prescribing patterns revealed concerning trends: only 31% of prescriptions were culture-guided, while broad-spectrum antibiotic utilization exceeded 70% in hospitalized patients. No fully implemented AMS programmes were identified across studies. Recommended interventions included point prevalence surveys (*n*=2), WHO AWaRe benchmarking (*n*=1), antimicrobial prescribing audits (*n*=3), plus educational initiatives and awareness campaigns.

**Conclusions:**

This systematic review shows that antimicrobial stewardship (AMS) in Bangladesh remains critically underdeveloped, with no fully implemented programmes identified. Empirical prescribing predominates (69% non-culture-guided), alongside excessive broad-spectrum antibiotic use (>70%). Major barriers include absent institutional policies and weak diagnostic infrastructure. Key recommendations are to establish mandatory AMS policies, create multidisciplinary teams, apply WHO AWaRe classification and strengthen laboratory capacity. Future research should focus on standardized outcomes, cost-effectiveness analyses and longitudinal evaluations to guide evidence-based policy and sustainable AMS implementation across Bangladesh’s healthcare system.
